# Retraction: Angiopoietin-Like 4 Confers Resistance to Hypoxia/Serum Deprivation-Induced Apoptosis through PI3K/Akt and ERK1/2 Signaling Pathways in Mesenchymal Stem Cells

**DOI:** 10.1371/journal.pone.0194448

**Published:** 2018-03-12

**Authors:** Meng Hou, Jinjin Cui, Jingjin Liu, Fang Liu, Rui Jiang, Kai Liu, Yongshun Wang, Li Yin, Wenhua Liu, Bo Yu, and the PLOS ONE Editors

Following publication of this article (1), concerns were raised regarding the presented data.

In Figure 4, the 1 ng panel incorrectly shows a duplicate of the 10 ng panel.

Figures 8A and 8D incorrectly show duplicate Western blots.

Additionally, the authors acknowledge the following errors and concerns:

In Figure 5, there was an error in statistical analysis of quantitative Western blot data, resulting in error bars that were too small.

In Figure 10C, the cytoplasmic cytochrome C in the control normoxic cells is higher than expected. MSCs of suboptimal condition were used and antibody was used at a dilution of 1/1000, instead of the manufacturer’s recommendation of 1/10000.

In Figure 10G, debris and artefacts in the imaging raise concerns regarding the quality of cells and reagents used.

In Figure 10E, band positioning raises concerns regarding the identity of the proteins.

Uncropped, raw blot images are no longer available for further evaluation.

In view of the concerns regarding the reliability of the results and the absence of the raw data images, the authors and *PLOS ONE* Editors retract this article. The authors wish to apologize to readers.

MH, JC, JL, FL, KL, YW, LY, WL and BY agree with the retraction. RJ could not be reached.
